# A 5-Pathway Signature Predicts Prognosis Based on Immune-Derived lncRNAs in Patients with Breast Cancer

**DOI:** 10.1155/2022/2906049

**Published:** 2022-12-12

**Authors:** Bo Liu, Nian Zhu, Huixia Huo, Junqi Long, Xinchan Ji, Jinmeng Li, Xujie Zhuang, Huina Wang, Lujia Li, Yuhaoran Chen, Wei Yang, Shuangtao Zhao

**Affiliations:** ^1^School of Software Engineering, Faculty of Information Technology, Beijing University of Technology, Beijing 100124, China; ^2^School of Mathematical and Computational Sciences, Massey University, Palmerston North, New Zealand; ^3^Affiliated Hospital of Weifang Medical University, Weifang 261041, Shandong Province, China; ^4^Department of Thoracic Surgery, Beijing Tuberculosis and Thoracic Tumor Research Institute/Beijing Chest Hospital, Capital Medical University, Beijing 101149, China

## Abstract

**Background:**

Currently, predictive models were not developed based on the signaling pathway signatures of immune-related lncRNAs in breast cancer (BRCA) patients.

**Methods:**

We selected unsupervised hierarchical clustering algorithm to classify patients with BRCA based on the significant immune-derived lncRNAs from the TCGA dataset. And different methods including ESTIMATE, ImmuneCellAI, and CIBERSORT were performed to evaluate the immune infiltration of tumor microenvironment. Using Lasso regression algorithm, we filtered the significant signaling pathways enriched by GSEA, GSVA, or PPI analysis to develop a prognostic model. And a nomogram integrated with clinical factors and significant pathways was constructed to predict the precise probability of overall survival (OS) of BRCA patients in the TCGA dataset (*n* = 1,098) and another two testing sets (*n* = 415).

**Results:**

BRCA patients were stratified into the PC (*n* = 571) and GC (*n* = 527) subgroup with significantly different prognosis with 550 immune-related lncRNAs in the TCGA dataset. Integrated analysis revealed different immune response, oncogenic signaling, and metabolic reprograming pathways between these two subgroups. And a 5-pathway signature could predict the prognosis of BRCA patients between these two subgroups independently in the TCGA dataset, which was confirmed in another two cohorts from the GEO dataset. In the TCGA dataset, 5-year OS rate was 78% (95% CI: 73–84) vs. 82% (95% CI: 77–87) for the PC and GC group (HR = 1.63 (95% CI: 1.17–2.28), *p* = 0.004). The predictive power was similar in another two testing sets (HR > 1.20, *p*  <  0.01). Finally, a nomogram is developed for clinical application, which integrated this signature and age to accurately predict the survival probability in BRCA patients.

**Conclusion:**

This 5-pathway signature correlated with immune-derived lncRNAs was able to precisely predict the prognosis for patients with BRCA and provided a rich source characterizing immune-related lncRNAs and further informed strategies to target BRCA vulnerabilities.

## 1. Introduction

Breast cancer (BRCA) is one of the most frequently diagnosed cancers, and leading cause of cancer death among women aged 20 to 59 years [1, 2]. Globally, it is estimated that nearly 2.3 million new BRCA cases representing 11.7% of all cancer patients were diagnosed, with over 685,000 deaths in 2020 [3]. BRCA is a heterogeneous tumor, and its etiology and pathological manifestations are significantly different in various regions [4]. And clinical research has shown that the molecular subtypes of BRCA are four distinct subtypes: Luminal A, Luminal B, HER 2^+^, and basal-like [5]. Fortunately, due to the advances in clinical diagnosis and targeted therapy, the breast cancer mortality has been dramatically reduced [6]. In early invasive breast cancer, mastectomy, modified radical mastectomy, or breast conserving surgery was selected for patients with BRCA, or neoadjuvant treatments might be recommended before surgery, and then adjuvant treatments were recommended after surgery, including chemotherapy, anti-HER2 therapy, other targeted therapy, endocrine therapy, bisphosphonates, and radiotherapy [7, 8], especially for triple negative breast cancer (TNBC) patients, treatments options include multimodal chemotherapy, immune checkpoint inhibitors, and antibody-drug conjugate treatment [9]. Notably, over the past few years, there have been exciting advances in targeted immunotherapy research for the distinct subtypes of breast cancers [10, 11]. Recently, immunotherapy offers unprecedented opportunities for effective treatment of malignant tumors because of the positive role of immune system in tumorigenesis, development, and treatment [12, 13].

Recently, long noncoding RNAs (lncRNAs) have received increasing attention due to their important roles in tumors [14]. LncRNAs are RNA transcripts with over 200 nucleotides that do not encode proteins [15]. However, lncRNAs can physically interact with DNA, miRNA, mRNA, or protein, and consequently regulate gene expression by epigenetic, transcriptional, or translational regulation [16]. Therefore, lncRNAs play an essential role in the occurrence, progression, and prognosis of cancer [17–19]. Recent studies showed that immune-derived lncRNAs took effect in different stages of tumor immunity, such as antigen presentation, immune activation, and immune cell infiltration [20, 21]. Thus, immune-derived lncRNAs have attracted wide attention [22]. Therefore, this difference in immune-infiltrating microenvironment is partly attributable to the expression of some immune-derived lncRNAs during cell growth, apoptosis, and invasion [11]. Recently, several studies have shown that a rich tumor immune microenvironment is associated with prognostic value in a subset of BRCA, which indicates the potential of immune-derived lncRNAs in assessing tumor immune cell infiltration [23]. However, there are no reliable model studies involving some pathways to predict prognosis in BRCA patients.

In this study, BRCA patients are classified into two subgroups: good cluster (GC) and poor cluster (PC) based on the expression profiling of the significant immune-derived lncRNAs by unsupervised clustering method. Then the differences of immune infiltration are evaluated by ESTIMATE and CIBERSORT algorithms, and enrichment pathways are targeted by immune-related lncRNAs and nonimmune-related lncRNAs with GSEA method. After that, a 5-pathway signature is developed with Cox regression method to independently assess prognosis, which could precisely predict the probability of survival between the GC and PC group in BRCA patients from The Cancer Genome Atlas Program (TCGA) dataset. The generalization ability of the model is verified on another two independent Gene Expression Omnibus (GEO) datasets. Finally, we established a prognostic nomogram model based on the 5-pathway signature and the clinicopathological risk factors in the TCGA dataset. The performance and clinical benefit of this nomogram were evaluated in another two GEO datasets to validate its accuracy and utility.

## 2. Methods

### 2.1. Data Source

We downloaded breast cancer clinical materials and gene expression data from TCGA (https://cancergenome.nih.gov/), and GEO (https://www.ncbi.nlm.nih.gov/geo/) datasets. Ultimately, 1,513 BRCA patients and 113 normal controls were collected, including 1,098 patients' samples and 113 normal controls from TCGA, 327 patients' samples from GSE20685 [24], and 88 cases from GSE20173 [25], respectively. The expression levels were normalized using the variance stabilizing *VST* transformation method of “*DESeq*2.” A list of immune-derived lncRNAs, immune cells, and immune pathways of breast cancer patients were downloaded from the Immunology Database and Analysis Portal (ImmLnc, https://bio-bigdata.hrbmu.edu.cn/ImmLnc/jt-download.jsp) [26].

### 2.2. Identification of Different Expression Genes and Enriched Signaling Pathways

Different expression genes (DEGs) were identified based on the standard of |log2(Fold Change)| > 1 and *q-*values <0.05 [27] by using R package “*DESeq*2.” And a total of 550 immune-related lncRNAs and 639 nonimmune-related lncRNAs with significant expression between the GC and PC group were obtained from 3,824 different expression lncRNAs between tumor and normal controls in TCGA data. Hierarchical cluster analysis was applied on the different expression genes and breast cancer patients using R package “*factoextra*.” All the targeted mRNAs of the significant lncRNAs were generated based on Pearson Correlation Coefficient (PCC, |*r*| > 0.4 & *p*  <  0.05), and these targeted genes were then analyzed by Gene Set Enrichment Analysis (GSEA) or Gene Set Variation Analysis (GSVA) to identify enriched signaling pathways from Molecular Signature Database (MSigDB) [28]. A cut-off of FDR *q*-value ≤0.05 was applied to select the most significantly enriched signaling pathways.

### 2.3. Estimation of Immune Infiltration

The Tumor Immune Estimation Resource (TIMER) (https://cistrome.shinyapps.io/timer/) produced the immune infiltrates in tumors, including CD4^+^ T cells, CD8^+^ T cells, B cells, macrophages, dendritic cells, and neutrophils for evaluating tumor-infiltrating immune cells [29]. Using the deconvolution algorithm CIBERSORT (https://cibersort.stanford.edu/), we inferred the infiltration of 22 immune cells subsets between the GC and PC group [30]. In addition, MCP-counter [31], EPIC [32], and ImmuCellAl [33] methods were also selected to evaluate infiltrating macrophages. The R package “*ESTIMATE*” was utilized to calculate the tumor microenvironment score, including the stromal score, immune score, ESTIMATE score, and tumor purity [34]. The correlation between immune infiltration and risk scores was calculated by using the Pearson correlation (Wilcoxon test) in all models.

### 2.4. Protein-Protein Interactions

The STRING (https://string-db.org/) is a database of known and predicted protein-protein interactions, including direct (physical) and indirect (functional) associations between proteins, on a global scale [35]. In this study, the target genes of immune-related DEGs and nonimmune-related DEGs in the GC and PC group were mapped to the STRING database to acquire a critical assessment and integration of protein-protein interactions.

### 2.5. Statistical Analysis

Through the R package “*survival*” (version 3.2–13), we calculated significant potential prognostic factors in the univariate Cox regression model, and then entered these factors into the multivariable Cox proportional hazard model. A nomogram was developed based on the results of the multivariable analyses. The calibration curves and discrimination were computed to evaluate the performance of the nomogram and its clinical utility using R package “*rms*” [36]. The area under the curve (AUC) of the receiver operating characteristic (ROC) curve and concordance index (C-index) were utilized to evaluate the predictive capacity of the predictive model. The least absolute shrinkage and selection operator (LASSO) regression method was adopted to identify the signaling pathways most associated with overall survival using the R package “*glmnet*.” The actuarial probability of overall survival (OS) was evaluated by Kaplan–Meier estimates, and difference was compared using the log-rank test. All statistical analyses were performed by R version 4.0.2 with several packages, with *p*  <  0.05 as statistically significant.

## 3. Results

### 3.1. Immune-Related lncRNA Profiling Classified Patients with Breast Cancer into Two Subgroups Correlated with Prognosis

**Figure 1:**
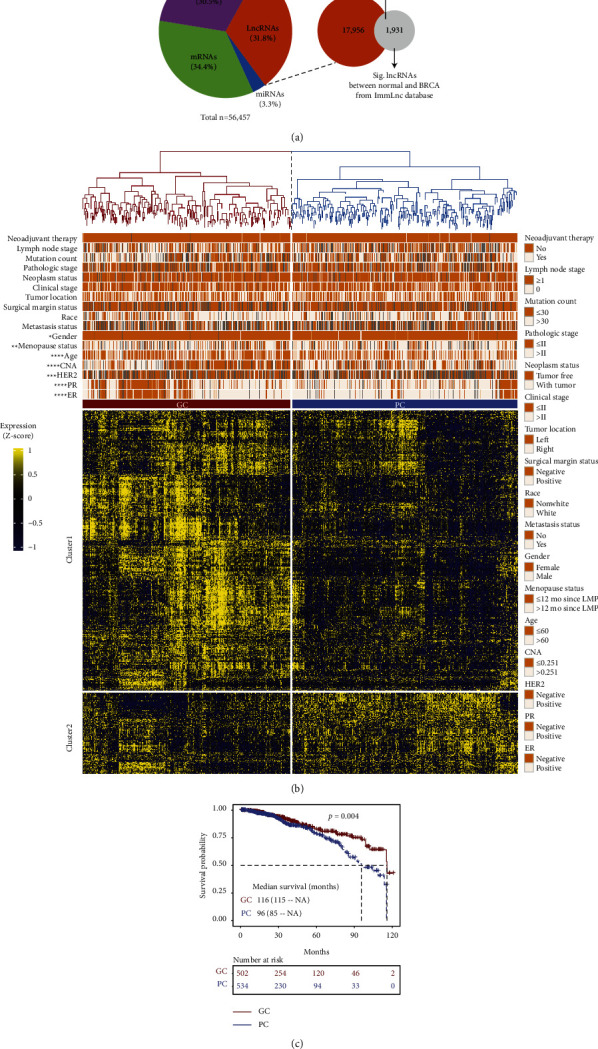
Explore the subgroups of breast cancer correlated with prognosis by immune-related lncRNAs. (a) The composition of genes from RNA sequencing involved in this study. (b) Heatmaps displayed the expression profiles of significant lncRNAs between the GC and PC group, *X*-axis denoted the differentially expressed lncRNAs (DElncRNAs), and *Y*-axis represented the breast cancer patients. The top tracks showed the histopathological characteristics. All the expression values were shown in line with the color scale. (c) Kaplan–Meier survival analysis between the two subgroups from the unsupervised hierarchical clustering. *p*=0.004 by log-rank test. GC, good-survival cluster; PC, poor-survival cluster. ^*∗*^*p* <  0.05, ^*∗∗*^*p* <  0.01, ^*∗∗∗*^*p* <  0.001, and ^*∗∗∗∗*^*p* <  0.0001.

The whole analysis process of this study is shown in Figure S1. A total of 17,956 lncRNAs and 19,408 mRNAs were collected from RNA-seq data in 1,098 BRCA samples and 113 normal samples. And the corresponding clinical data of 1,098 samples were downloaded from the TCGA dataset. We extracted 1,931 immune-related lncRNAs associated with immune cells and immune pathways in breast cancer patients from the ImmLnc database (Figure 1(a)). Then 3,824 lncRNAs were identified with significantly different expression between tumor (*n* = 2,725) and normal (*n* = 1,099) tissues (Figure S2). Of these DEGs, 550 genes were immune-related lncRNAs.

To explore the optimal clusters of 1,098 patients with breast cancer, we performed Principal component analysis (PCA) and hierarchical clustering analysis based on the expression profile of immune-derived lncRNAs which revealed two subgroups GC and PC (Figures 1(b) and S2B). In addition, a detailed analysis was conducted from the correlation between immune-related lncRNAs classification and clinical factors. Compared with the GC subgroup, patients in the PC subgroup had significantly older age, more positive status of ER/PR/HER2, more CNA (>0.251), and more menopause status with >12 months since LMP (Figure 1(b), Fisher test, *p*  <  0.05). However, the molecular subtypes were similarly distributed between the PC and GC group (Figures S3A and S3B), which were not significant prognosis across each subtype (*p*  >  0.05, Figures S3C and S3D). And we also observed a significant trend of shortened survival in patients in the PC subgroup (Figure 1(c), log-rank test *p* = 0.004). These results indicated the differences in clinical and histopathological phenotypes between two subgroups based on immune-related lncRNAs.

### 3.2. Immune-Cell Infiltration Analysis and Molecular Pathways between the Two Subgroups

**Figure 2:**
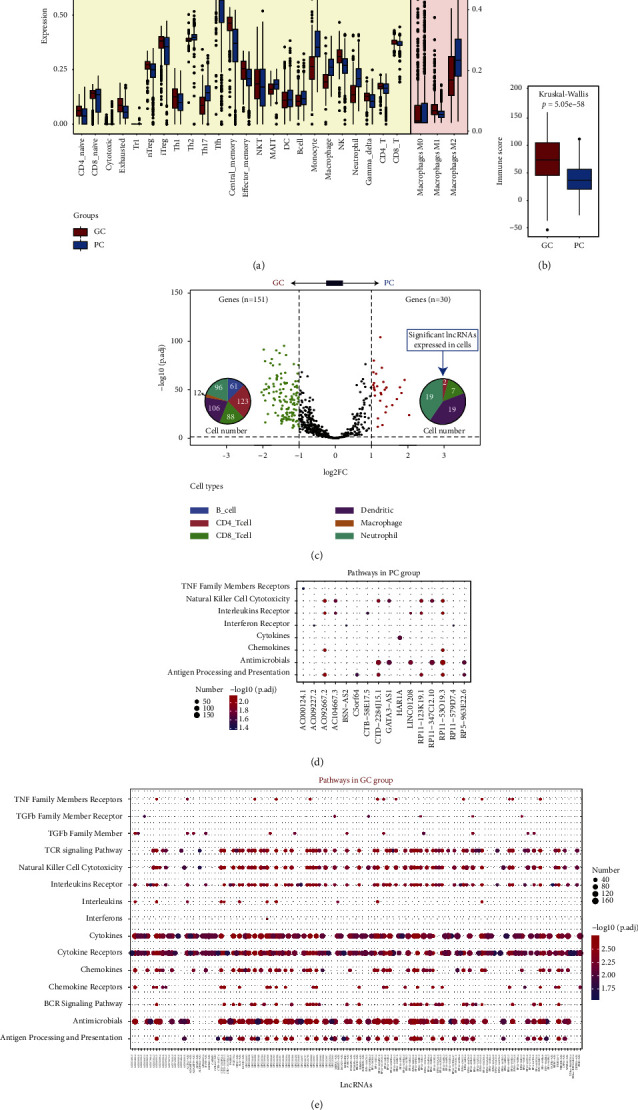
The immune composition between the two subgroups. (a) The difference of immune cells between the GC group (red) and PC group (blue) by ImmuCellAI and CIBERSORT algorithm. (b) The difference of the total immune score between the GC and PC group (Kruskal–Wallis test, *p*=5.05*e* − 58). (c) The volcano plot displayed the significant genes the between GC (*n* = 151) and PC (*n* = 30) group of breast cancer patients. Each red dot showed an up-regulated gene in the PC group but the green dot for the GC group. The distributed immune cells of these DEGs were represented with pie chart between these two subgroups. (d) and (e) Pathway enrichment analysis identified immune-related pathways enriched between the PC (d) and GC (e) group. DEGs, different expression genes, ^*∗*^*p* <  0.05, ^*∗∗*^*p* <  0.01, ^*∗∗∗*^*p* <  0.001, and ^*∗∗∗∗*^*p* <  0.0001.

To evaluate the immune-cell infiltration status, we performed six different algorithms including CIBERSORT and ImmuCellAI to quantify immune cell expression in breast cancer tissues. To clarify the intrinsic biological differences between the GC and PC group, we compared the immune cell composition of the TME (Figure 2(a)). Using ImmuCellAI to enumerate the abundance of immune cells subsets, we discovered that Th2, Th17, MAIT, B cell, monocyte, macrophage, and neutrophil cells were significantly up-regulated in the PC subgroup (*p*  <  0.05), but the other 17 immune cells in GC subgroup. Among 22 inferred immune cell types by CIBERSORT, the M2 macrophages were significantly increased in the PC subgroup but M1 in the GC subgroup. Generally, between the two distinct subtypes, the GC group exhibited a higher immune score because of a favorable prognosis (Figure 2(b), Wilcoxon Rank Sum Test, *p* = 5.05*e* − 58). Furtherly, we identified 181 immune-related lncRNAs with significant expression between two subgroups, of which 151 lncRNAs were mainly located in 123 CD4^+^ T cells and 106 dendritic cells from the GC subgroup but the other 30 lncRNAs most distributed in 19 dendritic cells and 19 neutrophil cells from the PC subgroup (Figure 2(c)).

Next, we performed pathway enrichment analysis to investigate dysregulated molecular processes informed by TCGA data. A total of 8 signaling pathways were identified within 16 immune-related lncRNAs in PC cluster (Figure 2(d)), such as natural killer cell cytotoxicity, TNF family members receptors, interleukins receptor, antigen processing and presentation, cytokines, chemokines, and antimicrobials. Using similar approaches, we analyzed the immune-related lncRNAs in GC cluster. A total of 15 signaling pathways were discovered, of which 8 common pathways were same with the ones above (Figure 2(e)). Taken together, the consistency between the immune profile and prognostic profile implied that our classification method was scientifically sound.

### 3.3. Dysregulated Molecular Pathways of Nonimmune-Related DEGs

**Figure 3:**
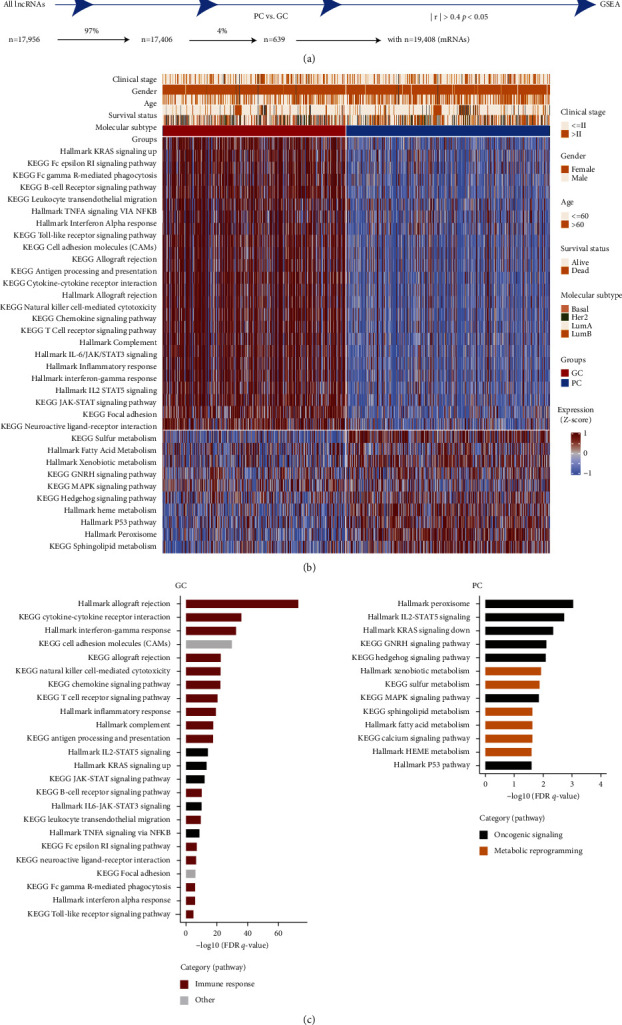
Pathway enrichment analysis of nonimmune-related lncRNA target genes. (a) Schematic flow chart showed the filtering process to identify the specific mRNAs correlated with nonimmune-related lncRNAs for the GSEA. (b) The heatmap displayed the significant pathways of the GSVA analysis between the GC and PC group. (c) Pathway enrichment analysis identified biological pathways enriched in the GC and PC subgroup. The curated gene sets were downloaded from the molecular signature database (MSigDB). Both the cancer Hallmark and KEGG gene sets were shown. The pathways were colored by their biological functions. FDR *q*-value, the *p*-value adjusted for the false discovery rate (FDR). A *q*-value threshold of 0.05 (5% FDR) was applied.

To explore the function of nonimmune-related DEGs between GC and PC subgroup, we performed a stepwise filtering process to identify the significantly enriched mRNAs (*n* = 19,408) correlated with nonimmune-related lncRNAs (Figure 3(a); *n* = 639; Pearson |*r*| > 0.4, *p*  <  0.05). Then pathway enrichment analysis was conducted to investigate dysregulated molecular processes of 2,318 mRNAs targeted by these nonimmune-related lncRNAs with significantly different expression between the GC (*n* = 534) and PC (*n* = 105) group. Then, a total of 34 signaling pathways were identified which were tumor specific, highly abundant, and significantly enriched in the GC (*n* = 24) and PC (*n* = 10) group (Figure 3(b)). The integrated analysis revealed enriched biological pathways including immune response and oncogenic signaling in the GC group but predominant composition of metabolic and oncogenic signaling in the PC group, such as the IL2-STAT5 signaling, hedgehog signaling pathway, MAPK signaling pathway, P53 pathway, xenobiotic, and sulfur metabolism (Figure 3(c)). These results demonstrated differences in signaling pathways based on nonimmune-related lncRNAs between two groups with different prognosis.

### 3.4. Correlation Analysis between Immune and Nonimmune-Related Composition in TME

**Figure 4:**
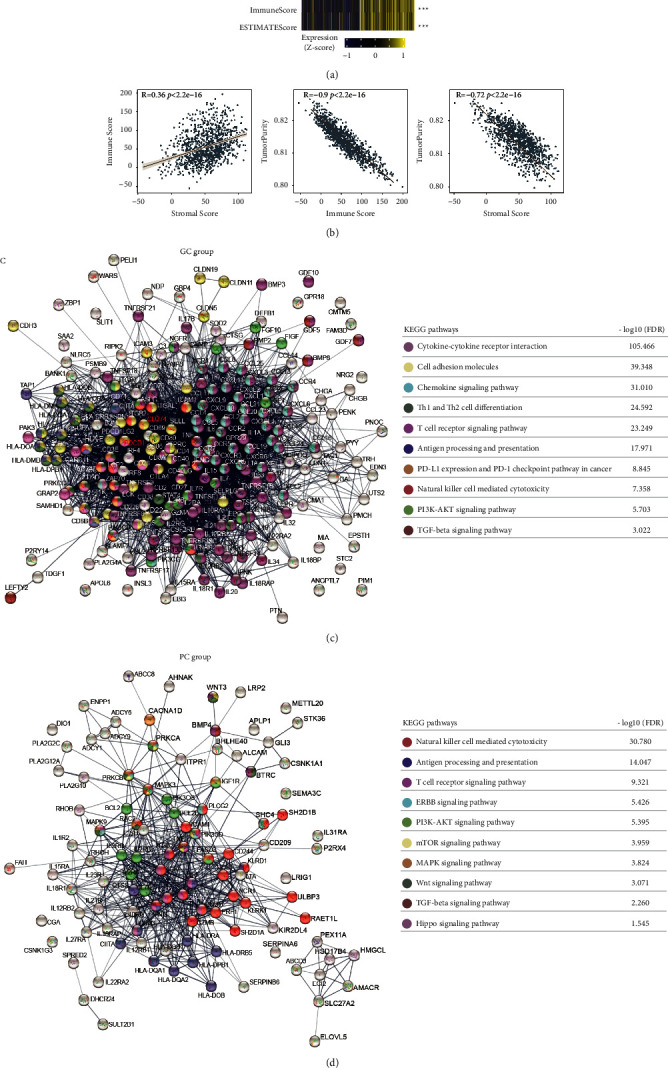
Landscape of intergroup crosstalk between immune and nonimmune signaling pathways. (a) Heatmap showed the immune and nonimmune composition between the GC and PC group. (b) Correlation analysis among the immune score, stromal score, and tumor purity. (c) and (d) The PPI network displayed the interaction between immune and nonimmune signaling pathways in the GC and PC subgroup, respectively. Network nodes represented the different proteins. Edges represented protein-protein associations. The line thickness indicated the strength of data support. DEGs, different expression genes.

To understand the relationship between the proportion of immune and stromal components in tumor microenvironment (TME), the ESTIMATE algorithm was to calculate the immune and stromal scores of BRCA tumor samples (Figure 4(a)). The results indicated that the immune score was positively correlated with the stromal score (*R* = 0.36, *p*  <  2.2*e* − 16) but negatively correlated with the tumor purity (*R* = −0.90, *p*  <  2.2*e* − 16), while the tumor purity was significantly negative in association with the stromal score (Figures 4(a) and 4(b); R = −0.72, *p*  <  2.2*e* − 16).

To better understand the interactions of target genes of the significant immune and nonimmune-related lncRNAs in TME, protein-protein interaction (PPI) networks were constructed using the STRING online tool. In PPI networks, proteins with similar functions tend to connect or interact with each other [35]. As a result, the network consisted of 10 modules, 223 nodes, and 3,083 edges in the GC group (Figure 4(c)). Similarly, there were 10 modules, 107 nodes, and 1,127 edges in the PC group (Figure 4(d)), and the *p*-values <0.001 showed that the PPI enrichment was of great significance (Figures 4(c) and 4(d)). Meanwhile, we observed that the targeted genes of immune and nonimmune-related lncRNAs in the GC group were mostly enriched in immune-related pathways such as the chemokine signaling pathway and the cytokine-cytokine receptor interaction pathway from the KEGG database (*p*  <  0.001), followed by oncogenic pathways such as the PI3K-AKT and the TGF-beta signaling pathways (*p*  <  0.001). But in the PC group, all the targeted genes were predominantly enriched in oncogenic pathways such as ERBB, mTOR, MAPK, TGF-beta, Hippo, and PI3K-AKT signaling pathways (*p*  <  0.001), while immune response pathways were secondary such as natural killer cell mediated cytotoxicity, antigen processing and presentation, and T cell receptor signaling pathway (*p*  <  0.001). Generally, these genes and signaling pathways were probably involved in the tumorigenesis and progression of breast cancer.

### 3.5. The 5-Pathway Signature Could Classify BRCA Patients into Two Groups with Significant Prognosis

**Figure 5:**
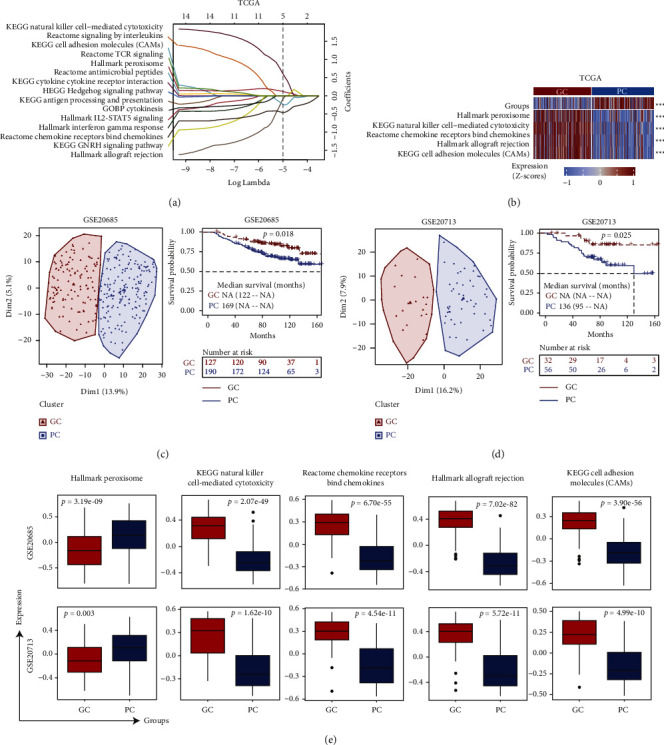
Identification of 5-pathway signature correlated with prognosis between the GC and PC group. (a) The coefficient profiles of the 15 candidate pathways from Lasso regression analysis in the TCGA dataset. (b) Heatmap plot of 5 candidate pathways selected by Lasso regression analysis associated with overall survival of breast cancer patients. (c) and (d) Kaplan–Meier survival analysis between two subgroups determined by the unsupervised hierarchical clustering in the datasets GSE20685 (c) log-rank test *p*=0.018 and GSE20713 (d) log-rank test *p*=0.025. (e) Box-plot showed a significant association of the signature pathways between the GC and PC group.

To establish a comprehensive and effective risk model for classification and prognosis prediction, we performed LASSO Cox regression analysis for the 15 signaling pathways from the immune and nonimmune-related enrichment. After 10-fold cross-validation, a total of 5 pathways were highlighted by the minimum partial likelihood deviance (Figure 5(a)), such as KEGG cell adhesion molecules (CAMs), KEGG natural killer cell-mediated cytotoxicity, Hallmark peroxisome, Reactome chemokine receptors bind chemokines, and Hallmark allograft rejection. And this 5-pathway signature could divide the breast cancer patients into two subgroups (GC and PC) with the significant prognosis based on the median score of these signatures (Figures 5(b) and S4). To confirm our discoveries, we selected another two GEO datasets to validate the prognostic power of 5-pathway signature. Similarly, we stratified the breast cancer patients of each independent cohort into two groups (GC and PC) by using PCA. In accordance with the results above from TCGA, patients with breast cancer from GSE20685 in the PC group (*n* = 197) had a higher risk (HR = 1.849, 95% CI: 1.10–3.09) than those in the GC group (*n* = 130), while 84% of the 5-year OS rates (95% CI: 79–89) in the PC group were significantly poorer than those of 92% (95% CI: 87–97) in the GC group (*p* = 0.018, Figure 5(c)). The 5-pathwaysignature-based classification of another cohort from GSE20713 (*n* = 88) also showed the similar results (Figure 5(d)). The HR (PC vs. GC group) in this cohort was 3.182 (95% CI: 1.09–9.27). In addition, the 5-year OS rates in the PC group were 75% (95% CI: 65–87%) significantly worse compared with 93% (95% CI: 84–99%) of the GC group in GSE20713 (*p* = 0.025, Figure 5(d)). Otherwise, compared with the PC group, the enrichment score was significantly lower for the Hallmark peroxisome pathway (*p* = 3.19*e* − 09), but up-regulated for the other four pathways in the GC group (*p*  <  0.001, Figure 5(e)), which was consistent with the results from the TCGA analysis above. All these results suggested that this 5-pathway signature could classify breast cancer patients with significant clinical outcomes, and function as an unfavorable biomarker.

### 3.6. The 5-Pathway Signature Predicted the Survival Probability Independently and Precisely by Integrating Other Clinical Factors

**Figure 6:**
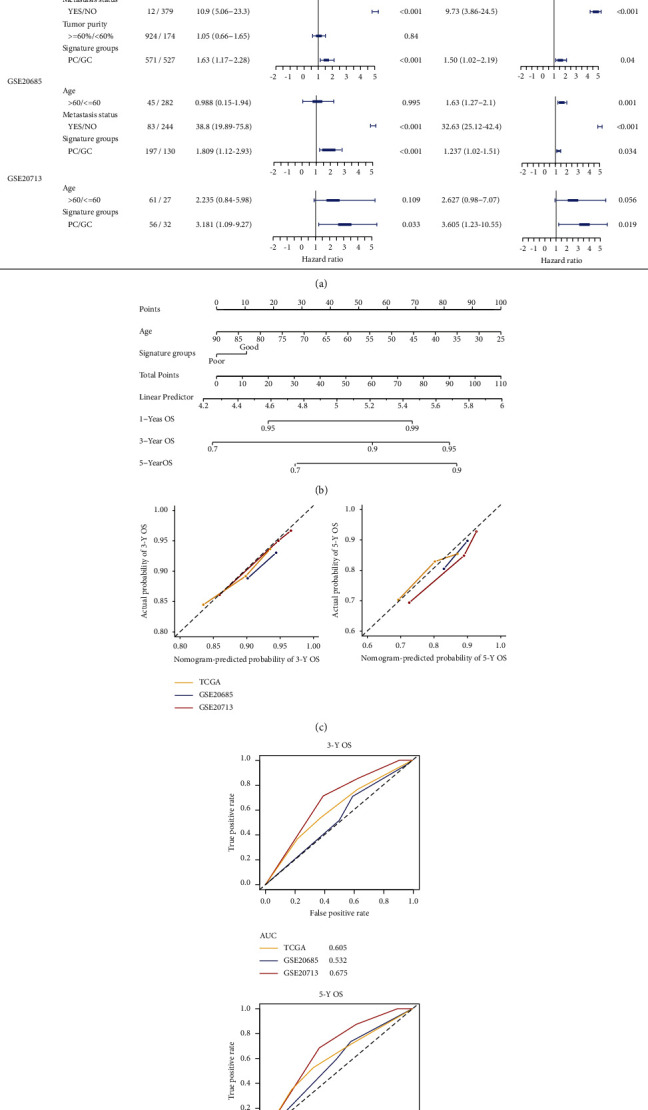
The 5-pathway signature could predict prognosis independently between the GC and PC group in TCGA and another two testing sets. (a) Univariable and multivariable cox analysis for the clinical characteristics and the 5-pathway signature in the TCGA and another two validation sets. (b) The nomogram for predicting overall survival of breast cancer patients. (c) Plots displayed the calibration of this 5-pathway signature according to the agreement of predicted 3 (left) or 5 (right)-year survivals. The plot showed the performance of the model relative to the 45-degree line, embodying perfect prediction. (d) Evaluation of the predictive power of the 3 or 5-year OS nomogram model between TCGA and the testing sets. HR, hazard ratio.

We performed univariate and multivariate Cox regression analyses to evaluate the independence of this 5-pathway signature in predicting prognosis, and discovered that this signature with 9 clinical factors including CNA, age, and metastasis status were significantly associated with survival (HR = 1.50, 95% CI: 1.02–2.19, *p* = 0.04; Figure 6(a)). Next, data stratification analysis was conducted among CNA, age, or metastasis status subgroups. As shown in Figure S5A, all patients were classified into the CNA ≤0.251 (*n* = 455) and >0.251 (*n* = 470) group, and the cutoff value of this signature could subclassify CNA >0.251 patients into the PC and GC group with significant prognosis (HR = 1.85, 95% CI: 1.13–3.04, *p* = 0.013), but in the CNA ≤0.251 group (*p* = 0.280). The 5-year OS rates of PC patients in CNA >0.251 group were 74% (95% CI: 66–83%), which was significantly lower than 80% (95% CI: 72–89%) of GC patients. Subsequently, the signature was further assessed in patients from different age or metastasis status (Figures S5B and S5C). The patients from each subgroup were significantly different prognosis (*p*  <  0.05) except for young subgroup (age ≤60 years). Also, we found that this 5-pathway signature was significantly associated with OS in another two external validation sets (GSE20685, HR = 1.237, 95% CI: 1.02–1.51, *p* = 0.034; and GSE20713, HR = 3.605, 95% CI: 1.23–10.55, *p* = 0.019; Figure 6(a)). These results indicated that the prognostic power of this signature was independent of other clinical factors in breast cancer patients.

We developed a nomogram combining the signature and one common clinical factor-age to explore a quantitative method for calculating the precise probability of clinical outcomes (Figure 6(b)). The 3-year and 5-year calibration plots showed that C-index from TCGA data (0.607, 95% CI: 0.577–0.637) was similar with that of another two validated datasets GSE20685 (0.576, 95% CI: 0.547–0.605) and GSE20713 (0.671, 95% CI: 0.637–0.704), which indicated that the nomogram worked well compared with an ideal model (Figure 6(c)). Further validation was performed when we applied ROC analysis into patients from the TCGA set and those two validated cohorts. The AUC values of 3 and 5-year nomogram were 0.605 (95% CI: 0.575–0.634) and 0.611 (95% CI: 0.581–0.641) in TCGA data, respectively, which were similar in another two validated datasets (Figure 6(d)). Generally, this nomogram could be utilized as a practical clinical tool to accurately predict the survival probability of breast cancer patients.

## 4. Discussion

BRCA is the most common type of malignant tumor, with high morbidity and mortality worldwide [2]. Breast cancer as a heterogeneous disease could be divided into different molecular subtypes including Luminal A, Luminal B, Basal, HER2^+^, and HER2^−^ based on the expression of estrogen receptor (ER), progesterone receptor (PR), and HER2 (Figure S3) [37–39]. In breast cancer systemic therapies, the prevalence and prognosis are utilized to manage these different breast cancer subtypes [39]. In addition, despite advanced diagnostic tools and treatment strategies, the recurrence rate of BRCA patients has not been significantly improved due to the lack of accurate and reliable biomarkers, which makes it difficult to identify early breast cancer and its subtypes [40]. In the occurrence, diagnosis and treatment of tumors, ncRNAs such as lncRNAs and miRNAs have become important markers [41, 42]. Therefore, more functional studies should be conducted on these immune-related lncRNAs, pathways, or tumor immune microenvironment, further to validate the predictive accuracy of breast cancer characteristics and discover potential immune-related mechanisms.

In this study, we first divided breast cancer patients into two subgroups (GC and PC groups) with significant prognosis by applying an unsupervised clustering algorithm. Then, we observed a statistically significant prognosis of patients between the two subgroups. The DEGs mainly involved in the immune response and oncogenic pathways might explain the potential difference of clinical outcomes between the GC and PC group. We then selected different algorithms to calculate the abundance of immune cells in the tumor microenvironment, and we discovered that patients who survived better were classified as a cluster with high immune infiltrates, which reflected the low degree of malignancy of patients and the favorable effects of various treatments. Among of them, in the CIBERSORT algorithm, we discovered that M2 macrophages were associated with poor survival of patients in the PC group, but M1 macrophages for favorable survival of patients in the GC group. Previous study demonstrated that M1 (activated; antitumoral) and M2 (alternatively activated; protumoral) phenotypes were significantly associated with distinct immunoregulatory functions [43, 44]. Then ImmuCellAI algorithm calculated the abundance of different immune infiltrating cells, and we inferred those 7 immune cells played a positive role in the PC group, such as Th2, Th17, MAIT, B cell, monocyte, macrophage, and neutrophil. Inversely, the other cells were enriched in the GC group with favorable prognosis, such as NK cells, CD4^+^ T cells, CD8^+^ T cells, and cytotoxic cells. Several previous studies have shown that NK cells cytotoxicity was mediated by both inhibitory and stimulatory receptors expressed on NK cells surfaces [45]. In addition, NK cells played a crucial role in the innate and adaptive immune systems [46, 47]. Likewise, we obtained similar results based on the ESTIMATE algorithm: immune infiltration was more abundant in the GC group. In the current study, research using endocrine therapy and targeted biological therapy has created new opportunities due to the improved understanding of immune escape of cancer cells and the discovery of selective immune checkpoint inhibitors [48]. Therefore, tumor immunology has become the fastest developing field in tumor research, and immunotherapy is the most promising treatment method in recent years.

Moreover, PPI network based on the targeted genes of DEGs displayed the interaction between immune and nonimmune signaling pathways in the GC and PC subgroup, respectively. These immune-derived DEGs were significantly associated with cytokine-cytokine receptor interaction and cell adhesion molecules pathway. On the contrary, nonimmune-related DEGs were correlated with the natural killer cell-mediated cytotoxicity pathway and antigen processing and presentation pathway [49]. In addition, LASSO regression revealed that five signaling pathways (Hallmark peroxisome, KEGG natural killer cell-mediated cytotoxicity, chemokines, Hallmark allograft rejection, and KEGG cell adhesion molecules (CAMs)) could be the main signaling pathways correlated with significant prognosis between the GC and PC group. Previous studies reported that these five signaling pathways were associated with cancer progression [50]. As a result, the GC group was with higher enrichment scores from the aspect of this pathway in this study.

Finally, we attempted to develop a predictive model for predicting the precise probability of clinical events by the significant clinical characteristics and this 5-pathway signature. Nomograms have become a standard prognostic tool in oncology research in predicting an individual's probability of a clinical event by using individual variables [51]. Moreover, a novel study reported that a pathway-based deregulation scoring matrix combined with the Cox regression and L1-LASSO regularization was to predict survival [52]. Consistent with previous studies, we identified two independent predictors (age and 5-pathway signature) embedded into the nomogram. In this study, the model had satisfactory predictive ability as the AUC of both training and validation sets was greater than 0.55. Then, the calibration analysis performed in training and validation sets revealed that 3 or 5-year survival predicted probability was similar to the actual probability. It is also inferred that this nomogram might stratify breast cancer patients into two clinical groups with different prognosis. All the results indicated that the 5-pathway signature could distinguish the survival prognosis of patients and reflect the level of immune response infiltration. This implied that the new nomogram would be clinically helpful for clinicians in tailoring a survival-associated treatment decision.

Furtherly, we also analyzed the composition and the survival status of five distinct breast cancer molecular subtypes in the subgroups, including Basal, Luminal A, Luminal B, HER2 positive (HR-positive), and HER2 positive (HR-negative) (Figure S3). Our results indicated that patients with Basal and HER2 positive (HR negative) showed poor survival. Therefore, after analyzing the survival status of these different BRCA subtypes, we believed that biomolecules might play different roles in the occurrence and development of these BRCA subtypes, especially related to immune processes and immune-related genes, which need to be further studied in the future.

The strength of the current study is that the immune-related signature was based on an online database, and each step of the screening had been tested for significance. Further, we analyzed the internal differences between the GC and PC group from the perspectives of tumor immune microenvironment, signaling pathways, protein-protein interaction, etc. The current study presented a novel immune-related prognostic approach for BRCA, thereby providing a new insight into the association between immune-related lncRNAs and survival in breast cancer patients. We realized that it would be great to reveal the potential lncRNA transcriptional mechanisms by examining the corresponding cancer cells and tumor tissues. However, we agreed that it is some weak in the biological validation in this study. In this study, we primarily focused on analyzing immune differences in subgroups and exploring a predictive signature to reveal the major functions of some lncRNAs in BRCA patients. Moreover, further efforts will be paid to validate these discoveries about the expression and function of these immune-related lncRNAs with modern empirical method in the next study.

## 5. Conclusion

This study revealed a novel finding by reporting a pathway-based classifier to predict prognosis of breast cancer in 1,098 patients, which was constructed by Cox proportional hazards regression algorithm with a 5-pathway signature filtered by LASSO survival regression. Clinical profiles analysis suggested that this classifier could add predictive power of clinicopathological features independently and had high sensitivity and specificity in predicting clinical outcomes. This study also extended immunology analysis to quantify the expression of immune cells in breast tumor tissue by applying different algorithms including CIBERSORT and ImmuCellAI. Moreover, a nomogram was developed for clinical practice that integrated this 5-pathway signature and age to predict precise survival rates for patients with breast cancer.

## Data Availability

The data generated and analyzed during the current study are available from the corresponding author on reasonable request.

## Abbreviations

BRCA: Breast cancer
TNBC: Triple negative breast cancer
OS: Overall survival
lncRNA: Long noncoding RNA
TCGA: The Cancer Genome Atlas
LASSO: Least absolute shrinkage and selection operator
C-index: Concordance index
PPI: Protein-protein interaction
GEO: Gene expression omnibus
PCA: Principal component analysis
GC: Good cluster
PC: Poor cluster
HR: Hazard ratio
95% CI: 95% confidence interval
ImmLnc: Immunology Database and Analysis Portal
GSEA: Gene set enrichment analysis
GSVA: Gene set variation analysis
MSigDB: Molecular signature database
FDR: False discovery rate
TIME: Tumor immune estimation resource
DEGs: Different expression genes
AUC: Area under the curve
ROC: Receiver operating characteristic
TME: Tumor microenvironment
KEGG: Kyoto Encyclopedia of Genes and Genomes
CAMs: Cell adhesion molecules
ER: Estrogen receptor
PR: Progesterone receptor
KM: Kaplan–Meier.

## Supplementary Materials

Supplementary Materials Supplement Figure S1: flow chart of study. Supplement Figure S2: filtering the significantly different lncRNAs between tumor and normal breast tissues for clustering analysis. Supplement Figure S3: the molecular subtypes distribution of breast cancer patients between the GC and PC group. Supplement Figure S4: the prognostic power for 5 signaling pathways in all the breast cancer patients stratified into two subgroups by the median value. Supplement Figure S5: Kaplan–Meier survival for all patients with breast cancer in TCGA data according to the model divided by the significantly clinicopathological risk factors. Supplement Figure S6: estimation of immune-cell Infiltration in the tumor microenvironment (TME) of the subgroups.
