# Supplementary material for: A 5-Pathway Signature Predicts Prognosis Based on Immune-Derived lncRNAs in Patients with Breast Cancer
Source: J Oncol. 2022 Dec 12;2022:2906049. doi: 10.1155/2022/2906049 (PMC9763012; doi:10.1155/2022/2906049)
Supplement: Supplementary Materials — Supplement Figure S1: flow chart of study. Supplement Figure S2: filtering the significantly different lncRNAs between tumor and normal breast tissues for clustering analysis. Supplement Figure S3: the molecular subtypes distribution of breast cancer patients between the GC and PC group. Supplement Figure S4: the prognostic power for 5 signaling pathways in all the breast cancer patients stratified into two subgroups by the median value. Supplement Figure S5: Kaplan–Meier survival for all patients with breast cancer in TCGA data according to the model divided by the significantly clinicopathological risk factors. Supplement Figure S6: estimation of immune-cell Infiltration in the tumor microenvironment (TME) of the subgroups. [file 2906049.f1.pdf]

# Supplementary Figures and Legends

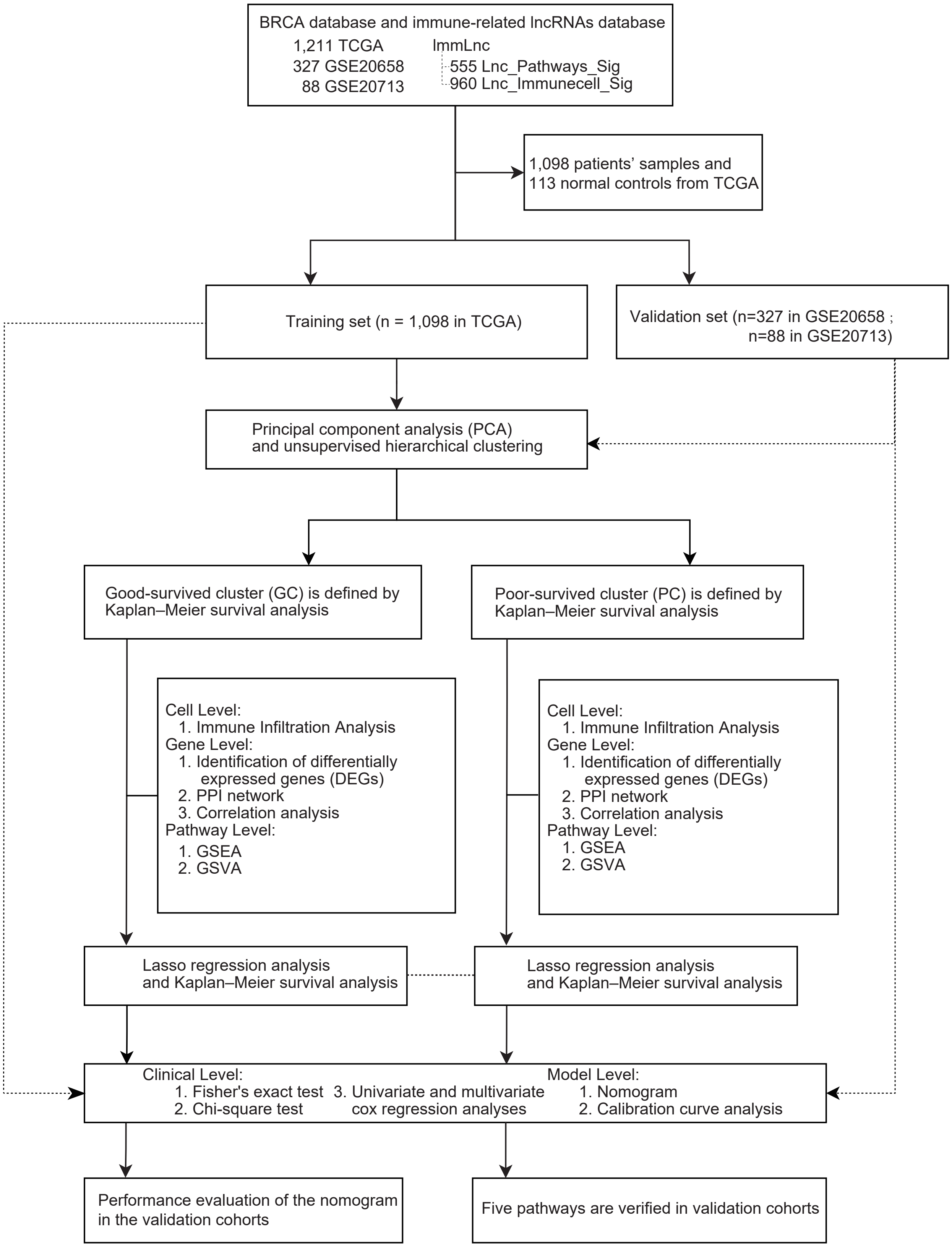


**Supplementary Figure 1. The flow chart of this study.** BRCA, Breast Cancer. TCGA, The Cancer Genome Atlas Program. GSEA, Gene Set Enrichment Analysis. PCA, Principal Component Analysis. GC, Good-survived cluster. PC, Poor-survived cluster. DEGs, Different expression analysis. GSVA, Gene Set Variation Analysis.

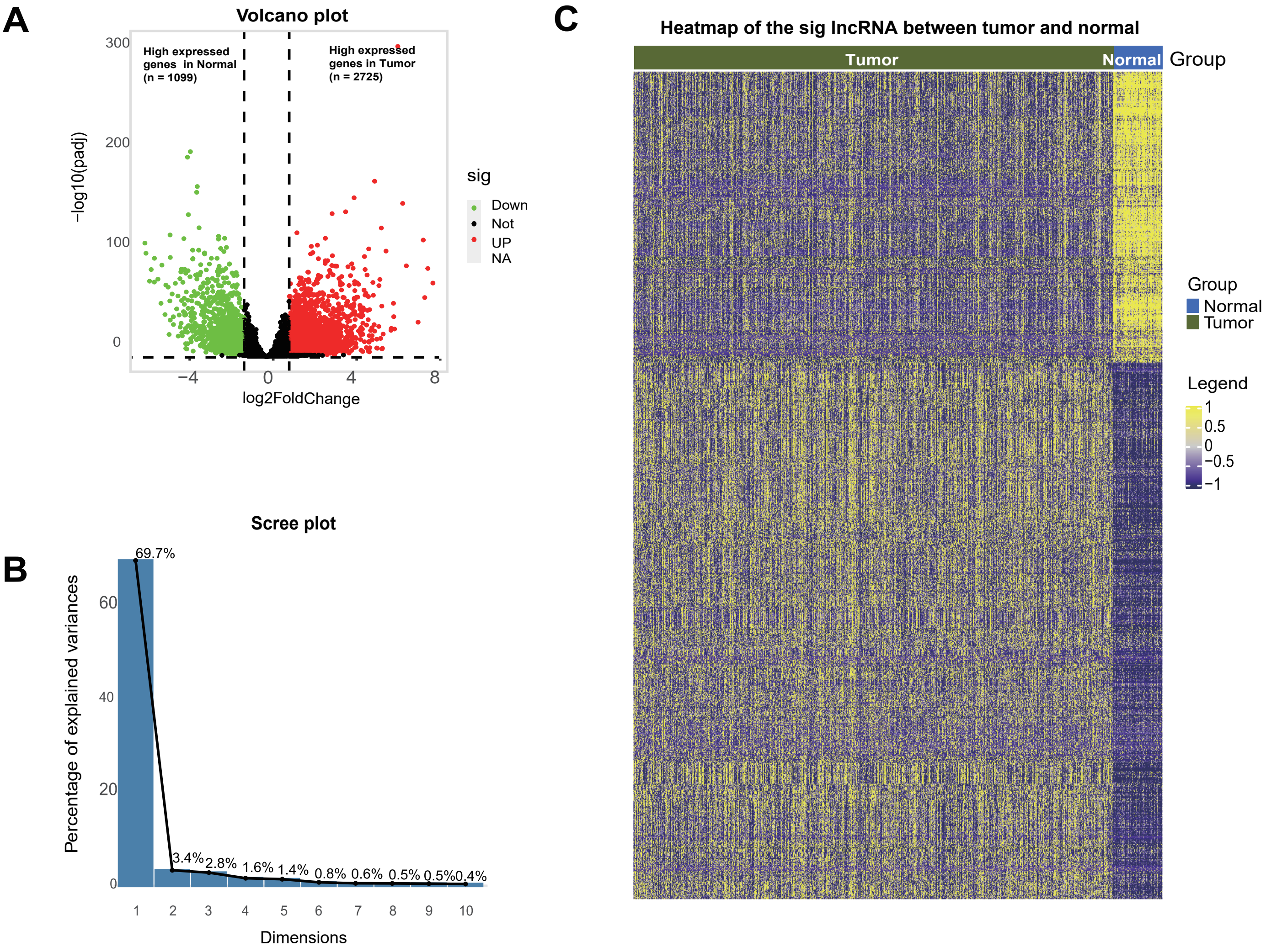


**Supplementary Figure 2. Filtering the significantly different lncRNAs between tumor and normal breast tissues for clustering analysis.** (A) The volcano plot displayed the DEGs between tumor and normal group. A cutoff fold change of ≥2 or ≤-2 and an FDR q-value were applied, and the significant genes were colored with green (high expression in normal tissue) or red (high expression in tumor tissue) in the plot. (B) The scree plot was to identify the final clusters with 3,824 DEGs in TCGA data. (C) Heatmap showed the DEGs (n=3,824) between normal and tumor tissues in TCGA data.

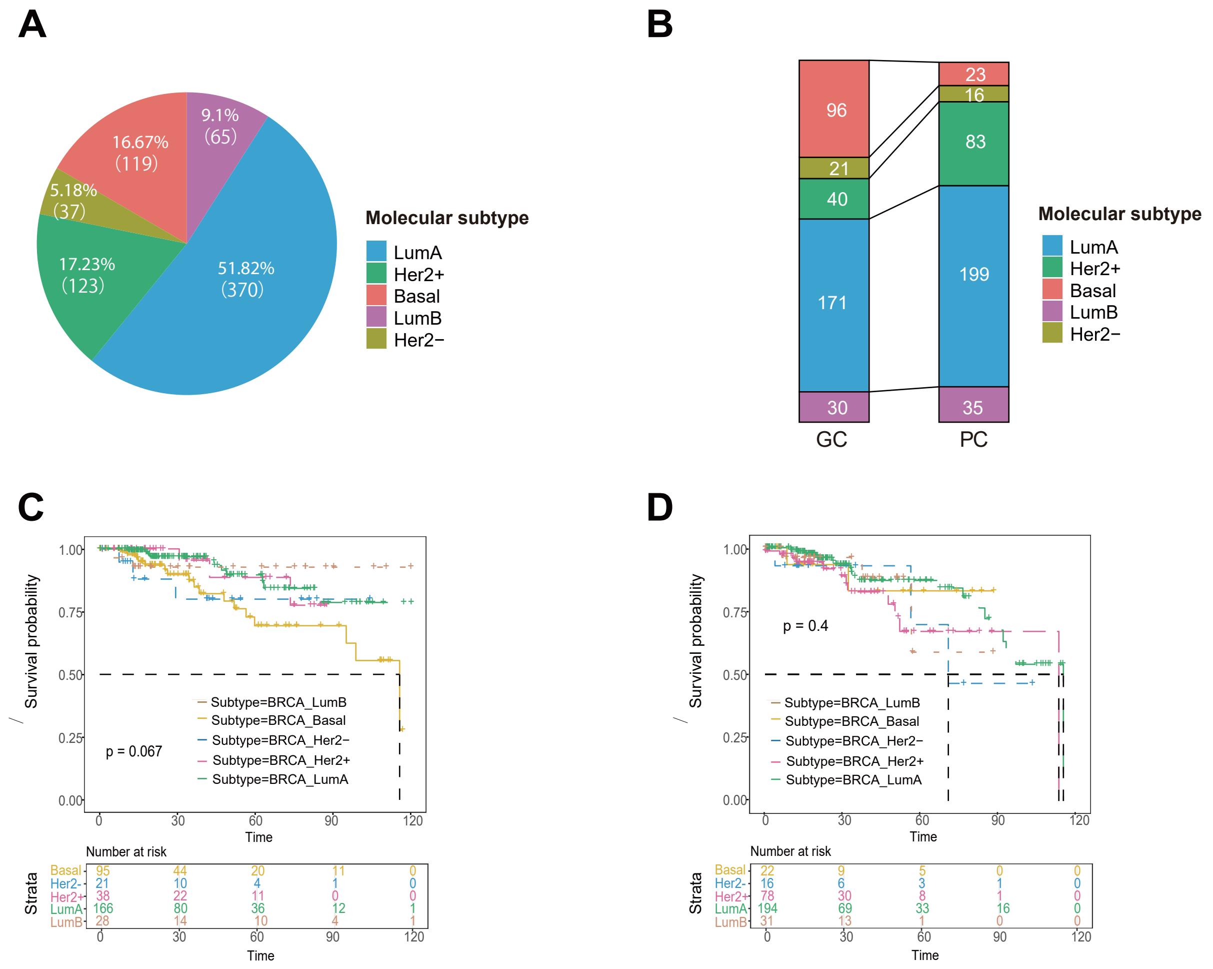


**Supplementary Figure 3. The molecular subtypes distribution of breast cancer patients between GC and PC group.** (A) Pie plot showed the composition of molecular subtypes in breast cancer patients. (B) The alluvival plot displayed the relationships of the molecular subytpes of breast cancer between GC and PC subgroup. (C) Survival analysis of the breast cancer patients across the molecular subtypes (D) Survival analysis of the breast cancer patients across the molecular subtypes in PC group (p = 0.400, log-rank test). LumA, Luminal A. LumB, Luminal B. Her2+, HER2 positive (HR-positive). Her2-, HER2 positive (HR negative). Basal, Basal-like.

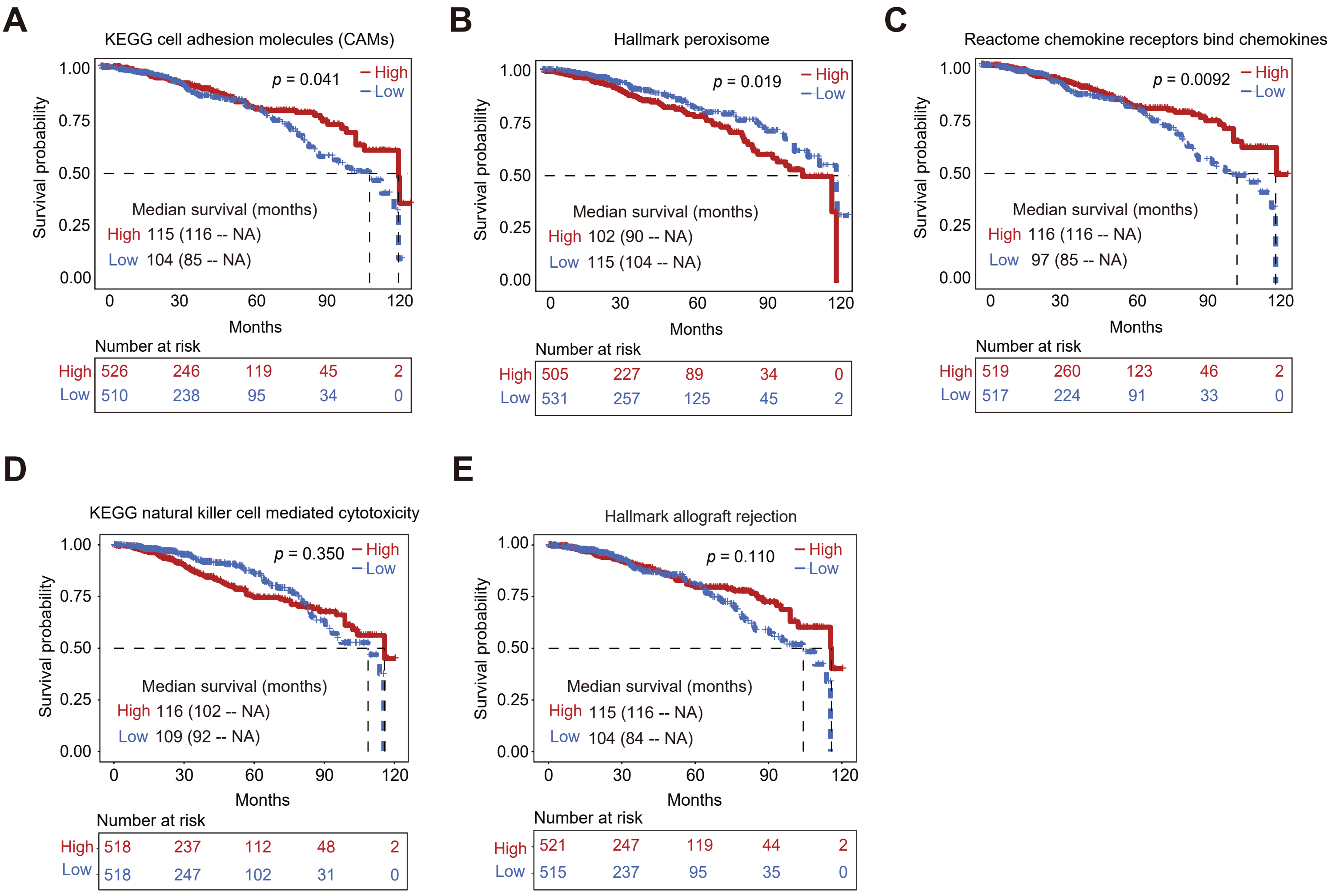


**Supplementary Figure 4. The prognostic power for 5 signaling pathways in all the breast cancer patients stratified into two subgroups by the median value.** (A) KEGG cell adhesion molecules (CAMs, p = 0.041, log-rank test). (B) Hallmark peroxisome (p = 0.019, log-rank test). (C) Reactome chemokine receptors bind chemokines (p = 0.0092, log-rank test). (D) KEGG natural killer-cell mediated cytotoxicity (p = 0.350, log-rank test). (E) Hallmark allograft rejection (p = 0.110, log-rank test).

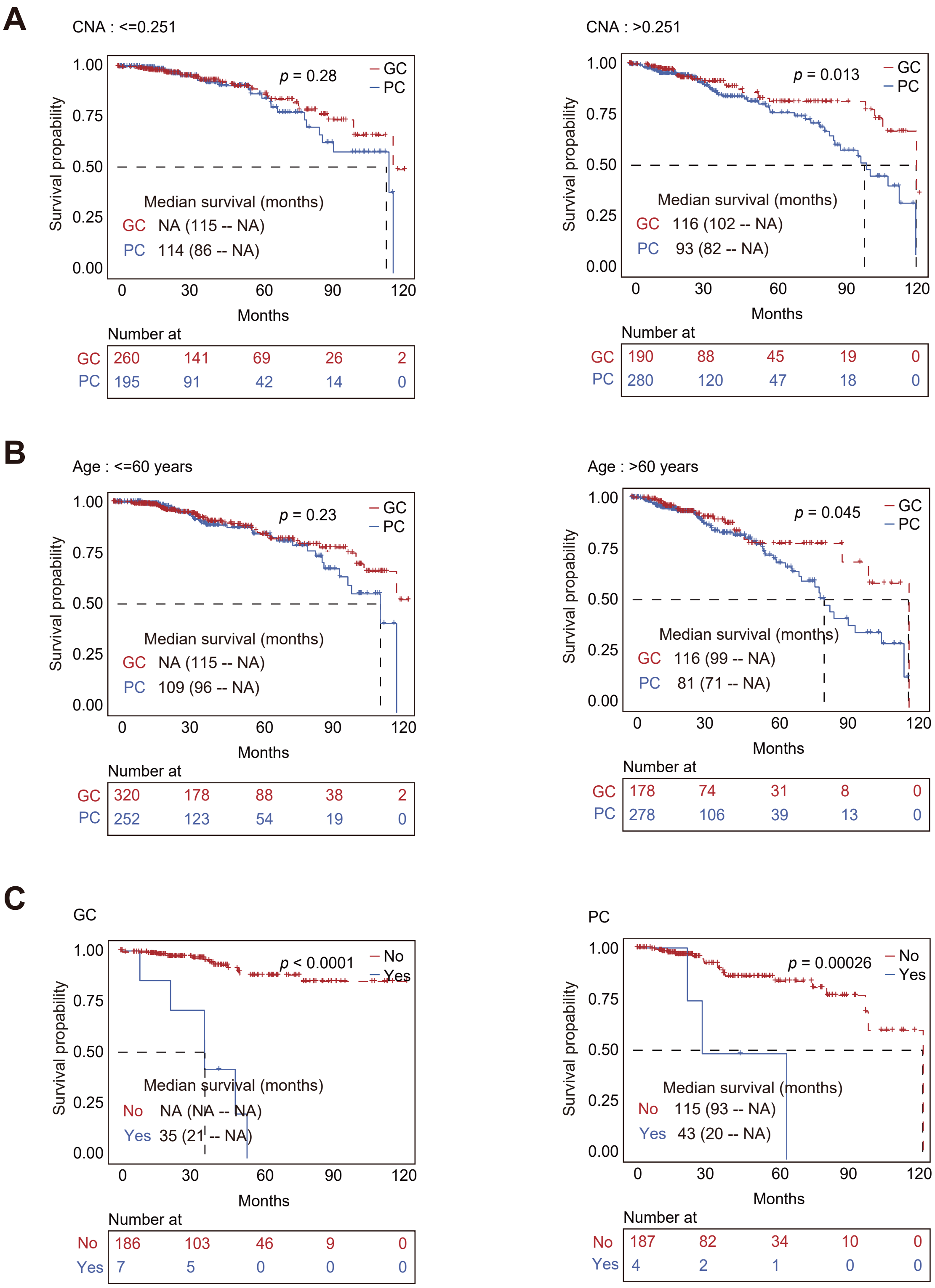


**Supplementary Figure 5. Kaplan-Meier survival for all patients with breast cancer in TCGA data according to the model divided by the significantly clinicopathological risk factors.** (A) CNA. (B) Patients' age. (C) The GC and PC subgroup.

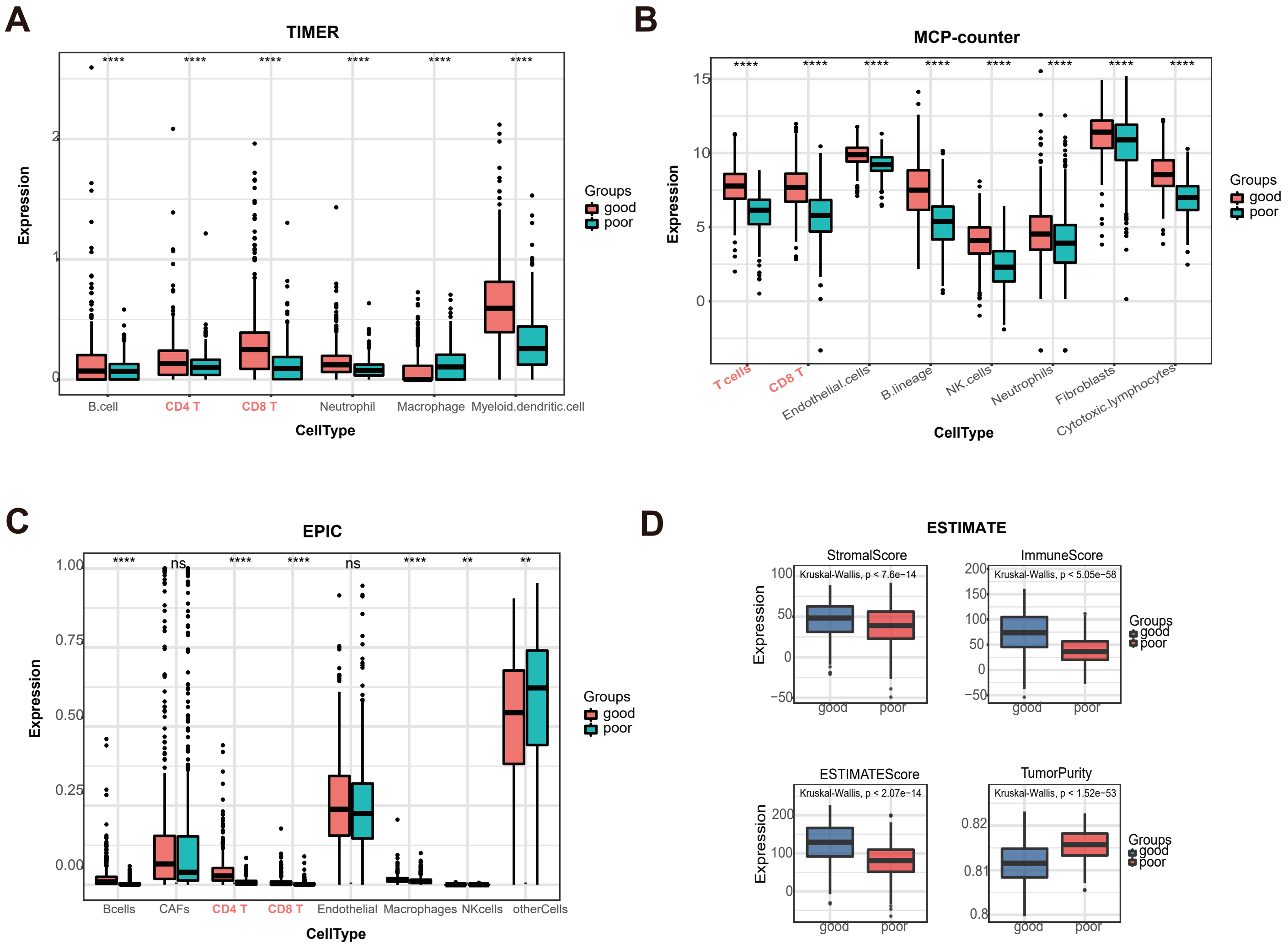


**Supplementary Figure 6. Estimation of immune-cell Infiltration in the tumor microenvironment (TME) of the subgroups.** (A) TIMER. (B) MCP-counter. (C) EPIC. (D) ESTIMATE.
